# Investigation of the Effects of Glabridin on the Proliferation, Apoptosis, and Migration of the Human Colon Cancer Cell Lines SW480 and SW620 and Its Mechanism Based on Reverse Virtual Screening and Proteomics

**DOI:** 10.1155/2023/1117431

**Published:** 2023-01-05

**Authors:** Tingting Li, Hang Li, Wenxin Xia, Mengyuan Li, Yang Niu, Xueyan Fu

**Affiliations:** ^1^Ningxia Medical University, Yinchuan, Ningxia 750004, China; ^2^Key Laboratory of Ningxia Minority Medicine Modernization, Ministry of Education (Ningxia Medical University), Yinchuan 750004, China

## Abstract

Colon cancer is a relatively common malignant tumor of the digestive tract. Currently, most colon cancers originate from adenoma carcinogenesis. By screening various licorice flavonoids with anticancer effects, we found that glabridin (GBN) has a prominent anticolon cancer effect. First, we initially explored whether GBN can inhibit proliferation, migration, and invasion and induce apoptosis in SW480 and SW620 cells. Next, we exploited reverse virtual and proteomics technologies to screen out closely related target pathways on the basis of a drug and target database. At the same time, we constructed the structure of the GBN target pathway in colon cancer. We predicted that GBN can regulate the phosphatidylinositol 3-kinase (PI3K)–protein kinase B (AKT)–mammalian target of the rapamycin pathway (mTOR) pathway to fight colon cancer. Finally, through Western blot analysis and qRT-PCR, we verified that the expression levels of the PI3K, AKT, and mTOR proteins and genes in this pathway were significantly reduced after GBN administration. In short, the promising discovery of the anticolon cancer mechanism of GBN provides a reliable experimental basis for subsequent new drug development.

## 1. Introduction

Colorectal cancer (CRC) is one of the common malignant tumors of the digestive tract. It ranks third among all malignant tumors in terms of incidence [1]. This disease is mainly located in the colon or rectum [2]. Thus far, the molecular mechanism of its pathogenesis has not been completely elucidated. Current scientific research shows that the main mechanisms of this malignancy involve the instability of the genome [3], the loss of calcium receptors in colonic epithelial cells [4], the effect of cell adhesion [5], and the abnormal expression of epidermal growth factors [6]. Moreover, many risk factors, including age, high-fat and high-protein diets, genetic factors, polyps, and adenomas, can lead to colon cancer [7]. At present, CRC is mainly treated through surgery combined with radiotherapy, chemical, and targeted drug therapy. However, these therapies can cause serious adverse reactions. Traditional Chinese medicine for the treatment of colon cancer involves tumor prevention and treatment; this approach can reduce the side effects or adverse reactions, such as gastrointestinal reactions, caused by radiotherapy and chemotherapy [8, 9].

Glabridin (GBN) is a flavonoid and one of the main active ingredients of *Glycyrrhiza glabra* L [10]. At present, GBN has a variety of pharmacological activities, including antiatherosclerosis [11], blood lipid reduction [12], nervous system protection [13], and anti-inflammatory [14] actions. In addition, it is commonly used to eliminate free radicals [15] and melanin [16]. GBN has been reported to have effects against liver cancer [17, 18], cervical cancer [19], and breast cancer [20]. Our research team found for the first time that GBN has a prominent anticolon cancer effect. On this basis, we used a variety of modern technologies to elucidate comprehensively the mechanism of GBN against colon cancer.

Computer reverse virtual technology can predict potential targets and signal pathways by scoring the binding energy of compounds and proteins [21]. The differential proteins of drug action can be found through proteomics technology. The signaling pathways involving differential proteins are identified to verify the target pathways screened by using virtual technology. In recent years, a growing number of scholars have successfully used these two techniques to study the mechanism of action of biology and drugs [22], which has provided great enlightenment for our research.

In this study, we combine the above two technologies to outline a pathway that is closely related to GBN's anticolon cancer activity. The mechanism of action of GBN may be related to the inhibition of the phosphatidylinositol 3-kinase (PI3K)–protein kinase B (AKT)–mammalian target of the rapamycin pathway (mTOR) signaling pathway. Finally, we experimentally verify the key indicators of proteomics and computer virtual technology results to clarify the mechanism of GBN in the treatment of colon cancer.

## 2. Materials and Methods

### 2.1. Reagents and Chemicals

GBN standard products were acquired from Shanghai Yuanye Biotechnology Co., Ltd. (batch number G006171216). Ammonia, iodoacetamide, and triethylammonium bicarbonate buffer were all procured from Sigma. Acetone and sodium lauryl sulfate were purchased from Shanghai Sinopharm. TMT 16Plex, Protein Ladder, Protease Inhibitor Cocktail, Pierce™ BCA Protein Assay Kit, NUPAGE 10% BT GEL 1.0MM 12 W, and Bond-Breaker™ TCEP Solution (TCEP) were produced by Thermo Fisher Scientific. Acetonitrile, methanol, formic acid, and other reagents required for the experiments were all of analytical grade. American Sigma Company provided dimethyl sulfoxide reagents. Fetal bovine serum (FBS) was sourced from Gibco, United States (10099-141). Beijing Soleibao Technology Co., Ltd., provided 0.25% trypsin–EDTA digestion solution and EDTA-free trypsin digestion solution.

### 2.2. Cell Culture

The human colon cancer cell lines SW480 and SW620 were purchased from Shanghai Luyu Biotechnology Co., Ltd. HT29 and HCT116 were provided by Procell Life Science & Technology Co. Ltd. All cells were cultured in L15 medium with 10% FBS. Double antibodies (100 *μ*g/mL streptomycin and 100 units/mL penicillin) were added to the incubator at 37°C and 5% CO_2_. The medium was changed every 2 days, and the cells were passaged at 1 : 3. Cells in the logarithmic growth phase were used in the experiment. The cells were digested and pipetted with 0.25% trypsin digestion solution (Beijing Soleibao Technology Co., Ltd., China). Glycyrrhizin was dissolved in dimethyl sulfoxide solution (Sigma, USA). The medium was diluted to the concentrations of 40, 20, 10, and 5 *μ*g/mL. The experiments were conducted in quadruplicate. In the blank experiment, dimethyl sulfoxide was used instead of glycyrrhizin.

### 2.3. Cell Proliferation Analysis

First, different colon cancer cells (SW480, SW620, HT29, and HCT116) in the logarithmic growth phase were digested and resuspended. Second, GBN was added at different concentrations (100, 50, 25, and 12.5 *μ*mol/L^−1^). After 24 h, 10 *μ*L of CCK-8 solution was added to each well, and the cells were cultured for 3.5 h. Finally, the absorbance value at 450 nm was measured with a microplate reader for calculation. The calculation formula was as follows: cell survival rate = [(As − Ab)/(Ac − Ab)] × 100% (As: experimental well OD value; Ac: control well OD value; Ab: blank well OD value).

### 2.4. Cell Apoptosis Analysis

The cell suspension (without trypsin–EDTA) was treated with different concentrations of GBN (20 and 40 *μ*g/mL). At the same time, 5 *μ*L of Annexin V-FITC was added. Subsequently, the cells were washed with PBS and suspended. Then, the cells were incubated with Annexin V-FITC/PI (Shanghai Beibo Biotechnology Co., Ltd.) at 37°C for 15 min in the dark. All data were analyzed by using a C6 flow cytometer (BD Biosciences, San Diego, CA, USA).

### 2.5. Nuclear Morphology Analysis

A cover glass was placed in a six-well plate, and colon cancer cells were implanted. The cells were treated with 40 *μ*g/mL GBN. After fixation and washing, 0.5 mL of Hoechst 33258 staining solution (Nanjing KGI Biotechnology Development Co., Ltd.) was added, and the cells were stained for 5 min. After sealing with a sheet, staining was observed with a fluorescence microscope (Olympus IX73), and images were collected.

### 2.6. Cell Migration Experiment

The cells were plated and incubated when their density reached 80%. Subsequently, the back of the plate was scratched, and the cells in treated medium containing 40 *μ*g/mL GBN were added. After the cells were cultured at 37°C for 0 and 24 h, photographs were taken and recorded to determine the healing distance of the scratches. Each experiment was repeated three times.

### 2.7. Cell Invasion Test

Cells in the logarithmic growth phase (density up to 80%) were placed in a Petri dish to prepare a cell suspension. A total of 200 *μ*L of cell suspension was added to the upper chamber of a Transwell plate. Then, 40 *μ*g/mL GBN-treated cells (without FBS) were added into the upper chamber and left to stand for 24 h. In addition, 500 *μ*L of 20% FBS medium was added into each well in the lower chamber of the Transwell. After 2 h, the Transwell chamber was washed twice with PBS and fixed with 4% paraformaldehyde for 30 min (upper chamber 200 *μ*L and lower chamber 500 *μ*L). Subsequently, after being washed two times with PBS, the cells were dyed with 0.1% crystal violet dye solution. Finally, an inverted microscope (Leica DMi1) was used for cell counting.

### 2.8. Western Blot Analysis

After GBN treatment, the colon cancer cells were collected and placed in prepared cold lysis buffer. Then, a BCA kit (Jiangsu KGI Biological Company, China) was used for concentration determination. Protein was separated by using sodium dodecyl sulfate-polyacrylamide gel electrophoresis (SDS-PAGE) then transferred to a polyvinylidene fluoride (PVDF) membrane (BioRad, Hercules, CA, USA). After blocking with 5% skim milk for 1 h, the membrane was incubated with the primary antibody overnight at 4°C and with the secondary antibody for 1 h at room temperature. Finally, the membrane was imaged with a chemiluminescence imaging instrument (GE Company, USA).

### 2.9. Quantitative Real-Time PCR

RNA was extracted by using a kit (Tiangen Biochemical Technology Co., Ltd., China). Reverse transcription was performed with a cDNA kit (Thermo Fisher Scientific Technology Company). Then, SYBR Green SuperMix (BioRad, Hercules, CA, USA) was used to detect the mRNA expression levels of caspase 3, caspase 9, Bax, and MMP2.

### 2.10. Reverse Virtual Screening of GBN against Colon Cancer

#### 2.10.1. Database Information

Computer reverse virtual screening technology (North Kedeyuan's reverse virtual screening platform: http://vslead.com/index.php=site/index) was used to perform molecular docking scoring between the chemical structure of GBN and the protein database of colon cancer diseases and cell lines. The targets and signal pathways involved in the curative effect of GBN were thus predicted.

The databases used for virtual screening included DGIdb, PubMed, PubChem, UniProt, PDB, DrugBank, SEA (http://sea.bkslab.org/), PAS (http://www.way2drug.com/PASSOnline/index.php), SuperPred (http://prediction.charite.de/index.php?site=home)/AutoDock Vina: (http://vina.scripps.edu/), KEGG database, and the NCBI website.

#### 2.10.2. Experimental Method

The target crystal structure, including 2119 active sites of 140 targets, corresponding to the disease genes was completely resolved by using the whole target library of the reverse virtual screening platform. The target structure corresponding to the disease gene was analyzed by using the part target library. These structures included 5487 active sites of 506 targets. The structural formula file of GBN was obtained by searching the DrugBank databank. Then, virtual molecular docking was carried out. The binding ability between the compound and protein was scored by applying the reverse virtual screening platform, and the top 200 results were returned. A high score indicated that the drug is bound to the target stably. Finally, target prediction and pathway analysis were carried out by comparing the results of the ProteinBank and KEGG databases.

### 2.11. Proteomics Analysis

#### 2.11.1. Protein Preparation and TMT Labeling

Tumor cells were lysed in lysis buffer at 70 Hz for 90 s. The lysed tissue sample was centrifuged, and the supernatant was collected. A BCA protein quantification kit (Thermo Fisher Scientific) was used to detect the protein content of the total protein extract of each group of tumor cells. After quantification, the protein sample was reductively alkylated with 10 mmol/L TCEP reducing agent (Sigma) and 40 mmol/L iodoacetamide (Sigma). Subsequently, the TMT 16Plex kit (Thermo Fisher Scientific) was used for labeling. The peptides in each group were labeled with different TMT labels (Table 1). Eventually, all the labeled samples were mixed, vacuum dried, and stored at −20°C for further analysis.

#### 2.11.2. High-pH Liquid Phase Separation and LC-MS/MS Analysis

The TMT-labeled peptides were dissolved in UPLC loading buffer. A reversed-phase C_18_ column ACQUITY UPLC BEH C18 Column (1.7 *μ*m, 2.1 mm × 150 mm, Waters, USA) was used for high-pH liquid phase separation.

Easy-nLC 1200 combined with a Q Exactive HF-X mass spectrometer was used to identify proteins. Peptides were dissolved in mass spectrometry loading buffer and separated on a C_18_ chromatographic column (75 *μ*m × 25 cm, Thermo, USA) after 120 min with a volume flow rate of 300 *μ*L/min. The EASY-nLC liquid phase was used as the gradient elution. Buffer phase A comprised 2% acetonitrile and 0.1% formic acid, and buffer phase B consisted of 80% acetonitrile and 0.1% formic acid. MS and MS/MS acquisitions were automatically switched. MS was performed with a full scan (m/z 350–1500). The acquisition mode was tDDA, and the cycle time was 2 s. Finally, TurboTMT was used to improve the resolution of the ion isotope.

#### 2.11.3. Protein Identification and Quantitative Analysis

The original files from the mass spectrometer were analyzed by using Proteome DiscovererTM Software 2.4. NCBInr database was used in the search with the false discovery rate of peptide identification set to FDR ≤ 0.01. Precursor mass tolerance was set to 20 ppm. Fragment mass tolerance was set to 0.02 Da and included at least one specific peptide. A protein with 1.2-fold change and significance of *P* < 0.05 was considered to be differentially expressed.

#### 2.11.4. Bioinformatic Analysis

Gene Ontology (GO: http://geneontology.org/) was selected for the functional annotation of all differential proteins from the three aspects of biological process, cell composition, and molecular function. The Kyoto Encyclopedia of Genes and Genomes (KEGG: http://www.genome.jp/kegg//) pathway database was used to analyze the metabolic and signal pathways involving differentially expressed proteins.

### 2.12. Experimental Verification

#### 2.12.1. Real-Time RT-qPCR

Table 2 shows all of the primers used for PCR and sequencing. First-strand cDNA was synthesized by using an RT kit (Thermo Fisher Scientific Technology Company). In accordance with the protocol provided in the instructions, 2 *μ*g of total RNA was extracted for reverse transcription, and the final reaction volume was 20 *μ*g. The PCR mixture contained 4 *μ*L of 5× reaction buffer, 1 *μ*L of Ribolock RNase Inhibitor (20 U/*μ*L), 2 *μ*L of 10 mm dNTP Mix, and 1 *μ*L of RevertAid M-MuLV RT (200 U/*μ*L) (Novazin Biotechnology Co., Ltd., China). All the PCR analyses were performed under the same conditions. The specificity of amplicons was verified through melting curve analysis (10 s at 95°C and 30 s at 60°C) after 40 cycles. The expression of the target gene relative to that of the internal control was calculated by using the 2^−△△^Ct method. Finally, the relative quantitative detection of the gene to be determined was performed.

#### 2.12.2. Western Blot Analysis

Proteins were extracted from human colon cancer cells and quantified as described in Section 2.11.3. An equal amount (20 *μ*g) of total protein in each sample was separated by using 10% SDS-PAGE. Then, the bands were transferred to a PVDF membrane (Millipore, MA, USA). Next, the membranes were blocked with 5% with skimmed milk powder at 37°C for 2 h and incubated with anti-PI3K (diluted 1 : 100), anti-Akt (diluted 1 : 100), anti-mTOR (diluted 1 : 100), and anti-*β*-actin (dilution 1 : 2000) antibodies overnight at 4°C. Hereafter, the membranes were washed with 0.5% Tween-20/PBS for 4 × 10 min, then incubated with the horseradish peroxidase-labeled goat antirabbit secondary antibody (1 : 2000) for 1 h. After four washes with PBST, the strips were exposed in a fully automated chemiluminescence instrument and stored. Protein expression level was defined as the gray value quantified with ImageJ software (National Institutes of Health, USA) and normalized to *β*-actin expression.

### 2.13. Statistical Analysis

All the experimental data were expressed as the mean ± SEM. Statistical analyses were performed with SPSS software (version 26.0, SPSS Inc., Chicago, IL, USA). The *t-*test (if *σ*1^2^ ≠ *σ*2^2^, then, the *t*-test was used) was performed to compare the means between the groups of samples. One-way ANOVA test (if *σ*1^2^ ≠ *σ*2^2^, then, Dunnett's *t-*test was used) was conducted to compare the means between multiple groups of samples, and *P* < 0.05 indicated that the difference was significant.

## 3. Results

### 3.1. GBN Inhibited the Proliferation of SW480 and SW620 Cells

**Figure 1:**
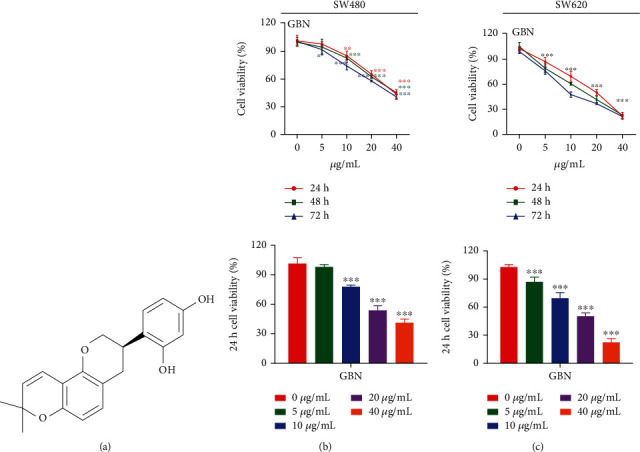
(a) Showing the structure of GBN. (b, c) Expressing the effect of GBN on the proliferation of SW480 and SW620 cells (*x̅*±*s*; *n* = 4). The effects of different concentrations of GBN for 24 h, 48 h, 72 h on the survival rate of SW480 and SW620 cells were determined (compared with the control group, ^∗^*P* < 0.05; ^∗∗^*P* < 0.01; ^∗∗∗^*P* < 0.001).

Figure 1(a) shows the chemical structure of GBN. The human colon cancer cell lines SW480, SW620, HT29, and HCT116 were selected to study the inhibitory effects of different concentrations of GBN (100, 50, 25, and 12.5 *μ*mol/L) on tumor cell proliferation. The results showed that different concentrations of GBN had a significant inhibitory effect on colon cancer cells (*P* < 0.05; *P* < 0.01). The findings in Table 3 confirm that the human colon cancer cell lines SW480 and SW620 were more sensitive to GBN than other lines and can thus be used for follow-up experiments. The survival rates of the SW480 cells treated with 40 *μ*g/mL GBN for 24 and 48 h were 43.75% ± 2.38% and 45.48% ± 0.23%, respectively (*P* < 0.001). The survival rates of each group of SW620 cells after 24 h of treatment with 20 and 40 *μ*g/mL GBN were 50.29% ± 1.73% and 22.16% ± 2.08%, respectively (*P* < 0.001). Cell proliferation in each treatment group significantly inhibited cell proliferation by varying degrees (Figures 1(b) and 1(c)) after 24 h of treatment relative to that in the control group. The difference among groups was statistically significant (*P* < 0.001). On the basis of these results, the follow-up mechanisms of action and proteomics experiments were performed with 20 and 40 *μ*g/mL GBN for apoptosis experiments and the action time of 24 h.

### 3.2. GBN Induced the Apoptosis of SW480 and SW620 Cells

**Figure 2:**
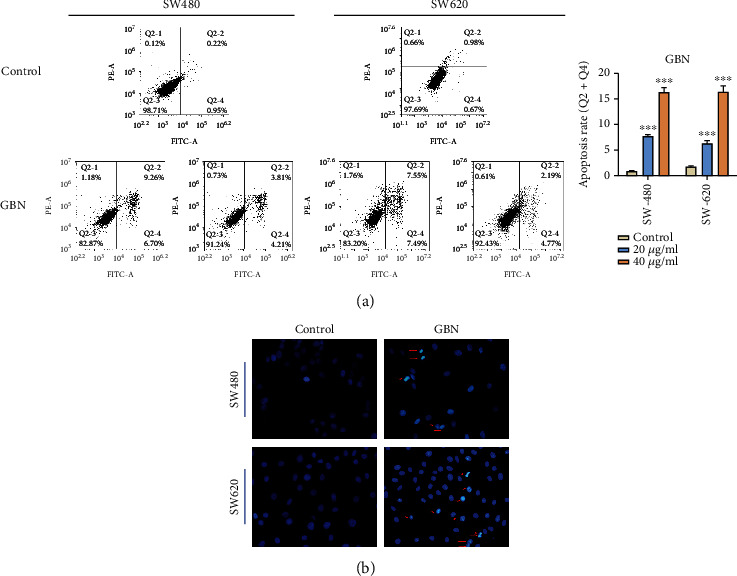
(a) The effect of 40 *μ*g/mL and 20 *μ*g/mL GBN on the apoptosis of SW480 and SW620 cells after 24 hours (compared with the control group, ^∗∗∗^*P* < 0.001). (b) The effect of 40 *μ*g/mL GBN on the nucleus morphology of SW480 and SW620 after 24 hours. The arrow in the figure showed pyknotic chromatin or broken nucleus.

The effects of GBN (40 and 20 *μ*g/mL) on the apoptosis of SW480 and SW620 were detected by using flow cytometry technology. The apoptotic rates of SW480 and SW620 cells under 20 *μ*g/mL GBN treatment were 7.77% ± 0.28% and 6.35 ± 0.53%. The apoptotic rates of SW480 and SW620 under 40 *μ*g/mL GBN treatment were only 16.35% ± 0.88% and 16.38% ± 1.17% (Figure 2(a)). Staining with Hoechst 33258 fluorescent dye revealed that treatment with 40 *μ*g/mL GBN induced different degrees of apoptosis in SW480 and SW620 cells. Cell morphology studies illustrated that nuclear pyknosis reduced and that nuclear fragmentation and incomplete cell fragmentation occurred (Figure 2(b)).

### 3.3. Effect of GBN on the Migration of SW480 and SW620 Cells

**Figure 3:**
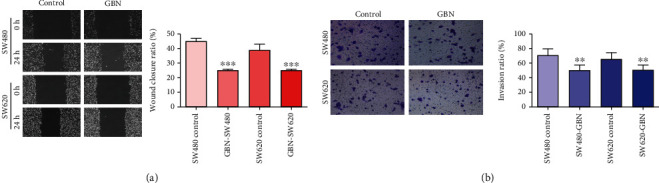
(a) After 40 *μ*g/mLGBN treatment of SW480 and SW620 cells, the healing of the cells at 0 h and 24 h was shown ($\overset{®}{x}\pm s$; *n* = 3) (comparison in the control group, ^∗^*P* < 0.05, ^∗∗^*P* < 0.01. ^∗∗∗^*P* < 0.001; 100×). (b) Effect of 40 *μ*g/mLGBN on the invasion ability of SW480 and SW620 cells ($\overset{®}{x}\pm s$; *n* = 3) (compared with control group, ^∗^*P* < 0.05, ^∗∗^*P* < 0.01. ^∗∗∗^*P* < 0.001; 200×).

The cell scratch experiment revealed that the migration rates of SW480 and SW620 cells under 40 *μ*g/mL GBN treatment were 34.10% ± 2.34% and 25.11% ± 0.73%, respectively. GBN significantly inhibited the migration of the human colon cancer cell lines SW480 and SW620 and decelerated the healing of scratches (*P* < 0.001) (Figure 3(a)).

### 3.4. Effect of GBN on the Invasive Ability of SW480 and SW620 Cells

The number of invasive SW480 cells in the GBN group was 51.00 ± 6.48. The number of invasive SW620 cells in the GBN group was 51.00 ± 6.56. GBN can significantly inhibit the invasive ability of SW480 and SW620 cells and significantly reduced the number of invasive cells compared with the control treatment (*P* < 0.01)(Figure 3(b)).

### 3.5. Effects of GBN on the Expression Levels of Proteins and Genes in SW480 and SW620 Cells

**Figure 4:**
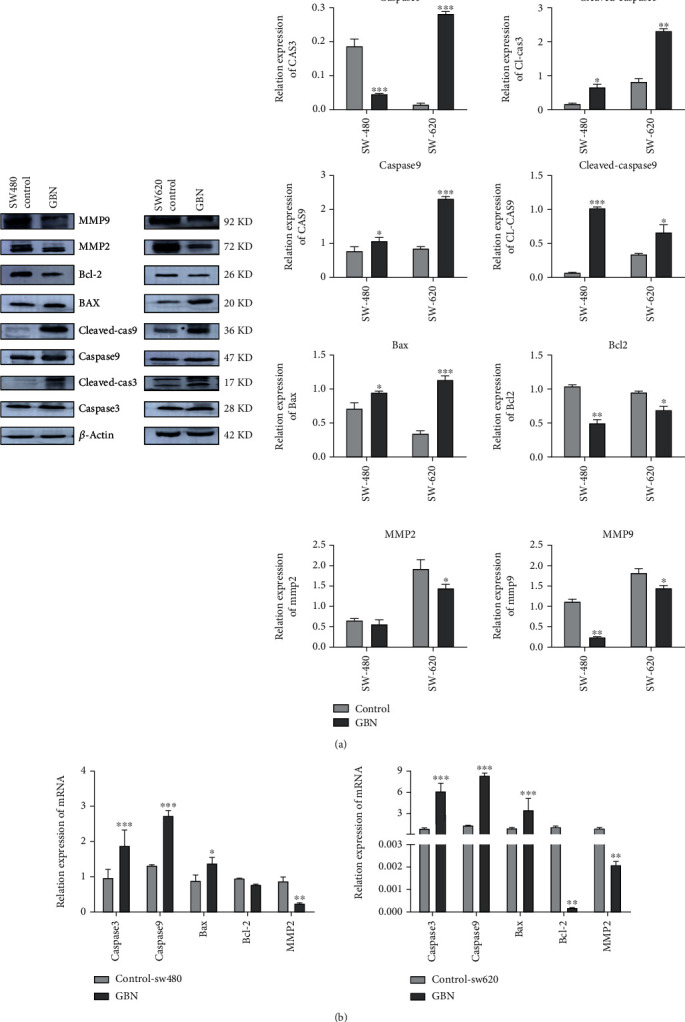
(a) The effect of GBN on the expression of apoptosis factor protein in SW480 and SW620 cells (compared with the control group, ^∗^*P* < 0.05; ^∗∗^*P* < 0.01; ^∗∗∗^*P* < 0.001). (b) The effect of GBN on the expression of apoptosis factor gene in SW480 and SW620 cells (compared with the control group, ^∗^*P* < 0.05; ^∗∗^*P* < 0.01; ^∗∗∗^*P* < 0.001).

In SW48 and SW6200 cells, the protein expression levels of Bax, cleaved-caspase 3, and cleaved-caspase 9 increased significantly after GBN drug intervention (*P* < 0.05, *P* < 0.001, and *P* < 0.001). Meanwhile, Bcl-2 and MMP9 protein expression decreased (*P* < 0.01) (Figure 4(a)).

Figure 4(b) also illustrates that the MMP2 gene level in SW480 cells significantly decreased (*P* < 0.01). In SW620 cells, the levels of Bcl-2 and MMP2 genes obviously decreased (*P* < 0.001). These results confirmed that GBN can promote the migration and invasion of SW480 and SW620 cell apoptosis through the caspase apoptosis pathway.

### 3.6. Establishment of a Disease Target Database to Screen the Target Results

A total of 18 potential targets were found in accordance with the virtual screening scoring results and the KEGG database analysis of the main related pathways of GBN involved in colon cancer (Table 4). KEGG database analysis revealed that GBN had six possible targets, namely, ABL1, INS, SRC, IRAK4, CHEK1, and KDR. This result confirmed that the virtual binding proteins of GBN and colon cancer were relatively concentrated in the PI3K–AKT, mTOR, and MAPK signaling pathways.

### 3.7. Candidate Target Determination for GBN

The screened GBN targets were compared with the CRC-related protein data obtained from a tumor genomics database (http://www.cbioportal.org). The results showed that GBN could upregulate 64 related proteins in CRC cells (Table 5). By comparing these 64 proteins with those in Table 4, we found that 13 candidate targets upregulated proteins in CRC cells and acted on signal pathways (Table 6). In addition, by using the above database, we found five candidate genes for the anticolon cancer effect of GBN, namely, INS, KDR, ABL1, SRC, and CHEK1. These proteins were mainly involved in the PI3K–AKT, mTOR, MAPK, and Wnt pathways.

### 3.8. Comparison of Screened GBN Targets with Genes Expressed in SW480 and SW620 Cells

First, we obtained the genetic information of SW480 and SW620 cell lines by using the Cancer Cell Line Encyclopedia. Then, through comparison with the screened targets presented in Table 5, we confirmed that the information of the five target genes in SW480 cells, including PTPN1, SEC14L2, PDE3B, PPARD, and TGFBR1, was consistent. Among these genes, PPARD and TGFBR1 were the same as the screened candidate target genes of GBN. Similarly, in the SW620 cell line, HSP90AB1 was the same as the screened candidate target gene of GBN. Finally, we confirmed that these target genes were involved in the Wnt, TGF-*β*, MAPK, and PI3K–AKT signaling pathways.

### 3.9. Proteomics Research and Analysis

**Figure 5:**
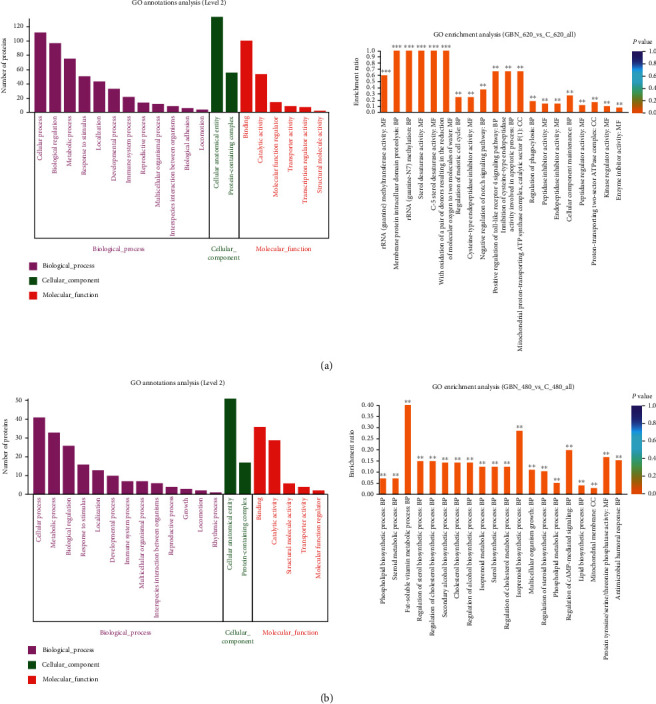
GBN differential protein GO classification map and enrichment map. Note: (a) The GO annotation picture of the different protein between GBN and the control group after acting on SW620 and SW480 cells. First, the figure showed the GO annotations of the top 20 abundances. Second, each column in the figure represented a secondary classification of GO. The height of the bar represented the amount of protein. Third, the abscissa represented the secondary classification term of GO, and the ordinate represented the number of proteins annotated to the secondary classification. Fourth, the three-color blocks represented the three branch types of GO.

We conducted proteomics analysis to obtain a global overview of GBN regulatory proteins and to further understand the mechanism of GBN's anticolon cancer action. High-pH RPLC analysis revealed that 7–20 amino acids were distributed in most of the peptides. This distribution pattern conformed to the general rules based on enzymatic hydrolysis and HCD fragmentation. By searching the NCBInr database, we identified 57 965 peptides and 6750 proteins. A protein with a fold difference that had increased by 1.2 times or decreased by 0.83 times and *P* < 0.05 was regarded as a differentially expressed protein. The SW620 cell control group (c_SW620) was compared with the administration group (GBN_620). A total of 150 proteins were differentially expressed between c_SW620 and GBN_620. The administration group had 43 upregulated proteins and 107 downregulated proteins. A total of 56 proteins were differentially expressed between SW480 cells (c_SW480) and the GBN group (GBN_480). Of these proteins, 22 were upregulated, and 34 were downregulated in the administration group (Figure 5(a)).

### 3.10. Bioinformatic Analysis of Differentially Expressed Proteins

We first performed GO enrichment analysis to clarify the biological importance of the differentially expressed proteins in c_SW620 and c_SW480 under GBN treatment. The functions of these proteins were annotated in accordance with cell composition, molecular functions, and biological processes (Table 7).

The GO annotation analysis of differentially expressed proteins between GBN_620 and the control group c_SW620 showed that 19 proteins were involved in biological processes, two were involved in cell components, and eight were involved in molecular functions. GO enrichment analysis revealed that the differentially expressed proteins had the highest degrees of enrichment in biological processes and molecular functions. When the enrichment of the differentially expressed proteins was corrected at *P* < 0.05, the enrichment rate was 100% (Figure 5(b)).

GO annotation analysis was performed on the differentially expressed proteins between the GBN_480 and c_SW480 group. These proteins were involved in 16 biological processes, two cell components, and seven molecular functions. GO enrichment analysis demonstrated that the differentially expressed proteins were enriched in the biological process function at the highest degree. The enrichment rate after concentration correction at *P* < 0.05 was approximately 40% (Figure 5(c)).

### 3.11. KEGG Functional Analysis of Differentially Expressed Proteins

**Figure 6:**
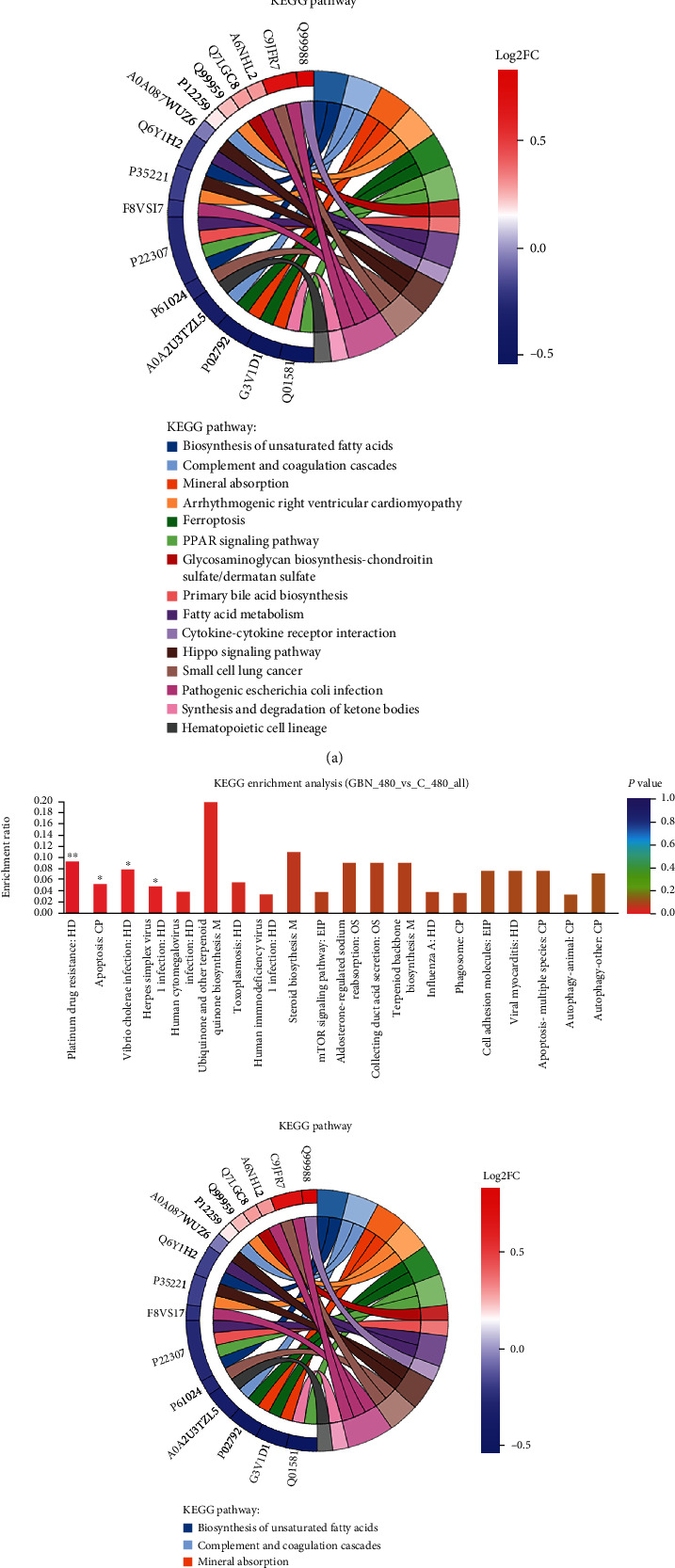
(a) GBN vs. c_SW620 differential protein KEGG enrichment analysis diagram; (b) GBN vs. c_SW480 differential protein KEGG enrichment analysis diagram. Note: (1) In the column chart, the abscissa represents the pathway name, and the ordinate represented the enrichment rate (the greater the ratio, the greater the degree of enrichment). (2) The color gradient of the column indicated the significance of enrichment, and the darker the color indicated the higher the enrichment degree of the KEGG term (^∗∗∗^*P* < 0.001, ^∗∗^*P* < 0.01, and ^∗^*P* < 0.05). The enriched string diagram showed the corresponding relationship between the target protein and the KEGG pathway. The left side was the protein, and the order from top to bottom showed log2FC. The greater the log2FC, the difference in the expression of the upregulated protein was the greater. On the right was the KEGG pathway name and *z* score of the target protein. On the right was the KEGG pathway name and *z* score of the target protein. Among them, count represented the total number of target proteins involved in this pathway. Up represented the number of upregulated proteins involved in this pathway. Down represented the number of downregulated proteins involved in this pathway. If *z* score was greater than zero, it meant that there were more upregulated proteins than downregulated proteins involved in this pathway, and this pathway was more likely to be activated. Conversely, *z* score was less than zero, which meant that this pathway is more likely to be inhibited.

By using the KEGG database, we can classify differentially expressed proteins in accordance with the pathways they participate in or the functions they perform. The analysis between groups revealed that in SW620 cells under GBN action, the differentially expressed proteins participated in KEGG pathways (the first 20 are listed in Table 8).  Table 9 illustrates that in secondary classification, differential protein pathways were mainly distributed in metabolic pathways, such as amino acids and nucleotides, translation, signal transduction, cell growth and death, and cancer-related pathways. We discovered that the highly enriched pathways with *P* < 0.05 were the unsaturated fatty acid biosynthesis pathway, p53 signaling pathway, mTOR signaling pathway, and other pathways (Figure 6(a)).

The analysis of GBN_480 and c_SW480 KEGG annotations showed that the differentially expressed proteins participated in 83 KEGG pathways (the first 20 are listed in Table 10). Secondary classification revealed that the differential proteins mainly concentrated in pathways related to energy metabolism, lipid metabolism, translation, signal transduction, transportation and catabolism, cell growth and death, cancer-related pathways, gene folding and degradation, and infectious diseases (Table 11). KEGG enrichment analysis illustrated that the pathways with *P* < 0.05, such as apoptosis and platinum drug resistance, were highly enriched (Figure 6(b)).

### 3.12. Differential Pathway and Protein Expression Analysis

**Figure 7:**
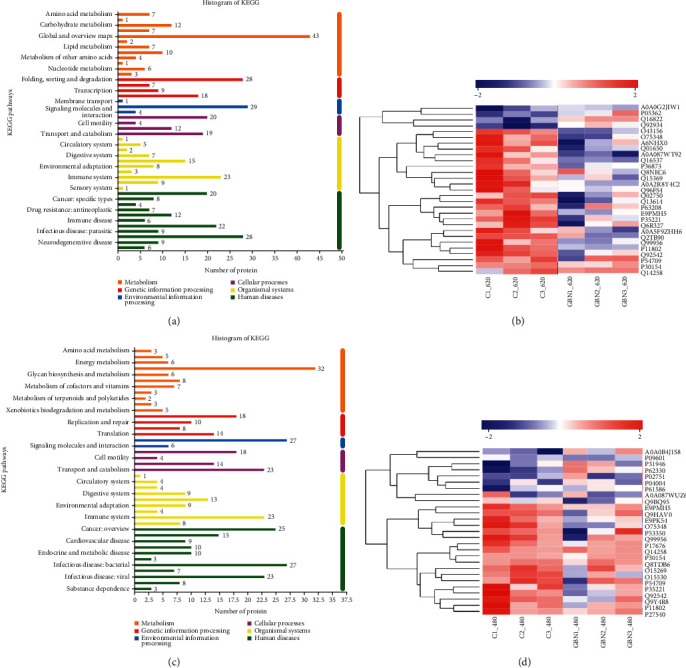
(a) Statistical histogram of pathway classification in SW620 cell administration group; (b) clustering heat map of 29 differential proteins in c_SW620 cell signaling pathway; (c) statistical histogram of pathway classification in SW480 cell administration group; (d) clustering heat map of 29 differential proteins in c_SW480 cell signaling pathway. Note: 7a and 7c represented 6 major categories and 44 subcategories of KEGG metabolic pathways of differential proteins common to the drug-administered group and the control group. 7b and 7d showed that each column represented a sample, and each row represented a protein. On the left was a dendrogram of protein clusters, and on the right was the name of the protein. The closer the two protein branches were, the closer their expression levels were.

We mainly investigated the metabolic pathways involving the key proteins to analyze the functional effects of differential proteins as a whole. We found a total of 382 differential proteins in c_SW620 administered with GBN. These proteins were involved in 260 KEGG metabolic pathways that were distributed in six major categories and 44 subcategories. In addition, pathway classification showed that the 29 differential proteins with the largest proportion were distributed in the signal transduction pathway in the environmental information processing category (Figure 7(a)). Next, through KEGG pathway analysis, we finally confirmed that in c_SW620 administered with GBN, the largest number of proteins participated in the two signaling pathways of mTOR and PI3K–Akt. Through the cluster analysis of 29 differential proteins, we found that the administration of GBN had a good callback effect on most signaling pathways (Figure 7(b)).

Protein levels changed significantly in c_SW480 cells administered with GBN. In the c_SW480 cell group, we found 348 differential proteins, which were involved in 225 KEGG metabolic pathways. The statistical results of pathway classification indicated that these pathways were distributed in six categories and 41 subcategories (Figure 7(c)). Among the differentially expressed proteins, 27 were distributed in the signal transduction pathway in the environmental information processing category. KEGG analysis indicated that that the largest number of proteins was involved in the two signaling pathways of cancer and PI3K–Akt. Cluster analysis indicated that the main downregulated proteins were serine protein kinase (P30154), guanine nucleotide-binding protein subunit-4 (Q9HAV0), fibronectin (P02751), 14-3-3*β*/*α* protein (P31946), Cyclin-dependent kinase CDK-4 (P11802), adhesion protein (P04004), 3-phosphoinositide-dependent protein kinase 1 (Q15530) telomere length regulatory protein TEL2 homolog (Q9Y4R8), V-type proton ATPase (O75348), and RhoA (P61586) (Figure 7(d)).

In short, the use of KEGG pathway enrichment analysis demonstrated that the largest number of proteins was involved in cancer, PI3K–Akt, mTOR, and other signaling pathways. After GBN acted together on the signal transduction pathway in SW620 and SW480 cells, the proteins that showed significant differences included NCSTN, V-ATP, CTNNA, CDK4, DUSP9, MKP4, ATP1B, and CD298.

### 3.13. Verification Experiment

#### 3.13.1. Real-Time qRT-PCR Analysis

**Figure 8:**
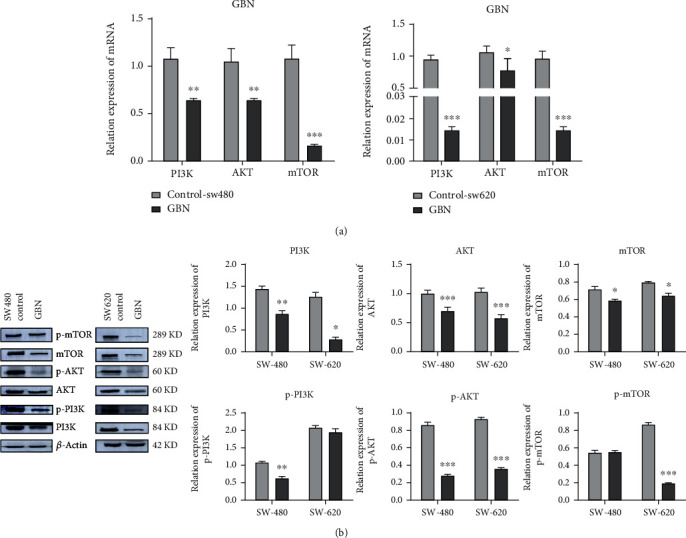
(a) Effect of GBN on the expression of PI3K–Akt–mTOR pathway-related proteins in colon cancer cells; (b) effect of GBN on the expression of PI3K–Akt–mTOR pathway-related genes in colon cancer cells (compared with control group, ^∗^*P* < 0.05; ^∗∗^*P* < 0.01; ^∗∗∗^*P* < 0.001).

The expression levels of PI3K, AKT, and mTOR genes in SW480 and SW620 cells under GBN intervention decreased significantly (*P* < 0.01, *P* < 0.01, and *P* < 0.001) relative to those in the control group (Figure 8(a)). The results suggested that GBN can inhibit the proliferation of human colon cancer SW480 and SW620 cells by regulating the PI3K–AKT–mTOR pathway.

#### 3.13.2. Western Blot Analysis

Comparison with the control group revealed that after GBN drug intervention, the p-PI3K/PI3K, p-AKT/AKT, and p-mTOR/mTOR protein phosphorylation levels in SW480 cells significantly decreased (*P* < 0.05, *P* < 0.001, and *P* < 0.001). The phosphorylation levels of p-PI3K/PI3K, p-AKT/AKT, and p-mTOR/mTOR proteins in SW620 cells decreased significantly (*P* < 0.001), as shown in Figure 8(b).

## 4. Discussion

Computer reverse virtual screening technology is often used to predict the signaling pathways and main targets of small-molecule compounds [23]. It has the advantages of low cost, fast speed, and high efficiency [24]. Our research group uses a system that is based on independent disease target databases (whole and partial) and molecular docking methods (AutoDock Vina). We also established a reverse virtual screening system with the help of the SDF format in the DrugBank database [25]. This system has been used by numerous researchers to verify the accuracy and practicality of anticancer targets [26–28]. Proteomics can detect the full set of proteins expressed by the complete genome in a cell [29, 30]. This technology can visually identify the regulation of drugs at the cellular protein level [31], then find targets and related pathways [32]. Therefore, on the basis of our experimental results, we confirmed for the first time that GBN can inhibit the apoptosis, migration, and invasion of SW620 and SW480 cells. At the same time, through computer reverse virtual screening technology and proteomics research, we predicted that GBN may inhibit the occurrence of colon cancer through the PI3K–AKT–mTOR pathway.

The PI3K–AKT–mTOR is closely related to the occurrence and development of CRC [33, 34]. PI3K is a key factor in this pathway [35]. Activated PI3K promotes the phosphorylation of PIP2 on the cell membrane into PIP3. The THr308 and Ser473 in the AKT protein are phosphorylated to bind with PIP3 [36]. PI3K activation can affect a variety of downstream effector molecules that can lead to reduced apoptosis [37], stimulate cell growth [38], and promote proliferation [39]. Activated Akt can also mediate apoptosis by regulating the expression of genes, such as Bcl-2 and caspase 3 [40]. mTOR, an important target gene downstream of Akt, is considered to be a member of the PI3K-related protein kinase family [41]. Activated mTOR can phosphorylate the translation repressor molecule eIF4E binding protein 1 and ribosomal protein p70S6K [33]. This process can inhibit tumor cell apoptosis [42]. Furthermore, the activated mTOR signaling pathway can increase the expression of matrix metalloproteinases to enhance extracellular matrix degradation. Therefore, this process can enhance tumor cell invasion and promote tumor cell migration [43]. Similarly, a large number of studies have reported that the PI3K–AKT–mTOR pathway can inhibit the growth, proliferation, and invasion of colon cancer cells [44]. Therefore, through Western blot analysis and qRT-PCR experiments, our team verified that GBN can exert its mechanism of drug action through the PI3K–AKT–mTOR pathway.

## 5. Conclusions

GBN has a certain application value because of its various anti-inflammatory, antibacterial, antitumor, anticardiovascular disease, and nervous system protective effects. Although it is currently not used as a clinical drug, it has an important curative effect without obvious side effects. Therefore, it is a highly promising potential drug. In summary, we used the combination of computer reverse virtual and proteomics technologies to study the mechanism underlying GBN's anticolon cancer effect. GBN can inhibit the proliferation of the human colon cancer lines SW480 and SW620 in vitro and induce tumor cell apoptosis. Its mechanism of action may be related to the inhibition of the PI3K–AKT–mTOR signaling pathway. Our research results demonstrated that the combination of computer reverse virtual and proteomics technologies is an effective tool for evaluating and exploring the efficacy and mechanism of action of Chinese medicine. At the same time, the results of this study provide a new understanding of how GBN inhibits proliferation and migration and promotes apoptosis in the human colon cancer cell lines SW480 and SW620 in vitro and evidence for GBN as a potential candidate drug for colon cancer.

## Figures and Tables

**Table 1:** List of sample marking order of each group.

Group one (SW620)		Group two (SW480)	
Mark	Sample	Mark	Sample
TMT16-126	CK1_1	TMT16-126	CK2_1
TMT16-127N	CK1_2	TMT16-127N	CK2_2
TMT16-127C	CK2_3	TMT16-127C	CK1_3
TMT16-128N	GBN1_1	TMT16-128N	GBN2_1
TMT16-128C	GBN1_2	TMT16-128C	GBN2_2
TMT16-129N	GBN2_3	TMT16-129N	GBN1_3
TMT16-134	Ref1	TMT16-134	Ref2

**Table 2:** Primer list.

Gene name		Sequence
*β*-Actin	F	5′-GGG AAA TCG TGC GTG ACA TTA AG-3′
	R	5′-TGT GTT GGC GTA CAG GTC TTT G-3′

PI3K	F	5′-CTT TGC GAC AAG ACT GCC GAG AG-3′
	R	5′-GCC TGA AGC TGA GCA ACA TCC TC-3′

AKT	F	5′-GCA GGA TGT GGA CCA ACG TGA G-3′
	R	5′-GCA GGC AGC GGA TGA TGA AGG-3′

mTOR	F	5′-CTT GCT GAA CTG GAG GCT GAT GG-3′
	R	5′-CCG TTT TCT GGG CTG GCT CTC-3′

**Table 3:** Effect of GBN on the survival rate of different human colon cancer cell lines($\overset{®}{x}\pm s$; *n* = 4).

Drugs	Concentration	SW480	SW620	HT29	HCT116
Blank group		102.81 ± 1.30	101.90 ± 0.72	101.04 ± 1.55	100.27 ± 0.67

Solvent control group	0.1% DMSO	99.95 ± 1.88	101.24 ± 2.37	99.37 ± 3.11	103.27 ± 3.07

GBN	100 *μ*mol·L^−1^	2.90 ± 1.01^∗∗^	13.34 ± 0.97^∗∗^	12.05 ± 0.68^∗∗^	4.64 ± 1.00^∗∗^
	50 *μ*mol·L^−1^	19.20 ± 2.48^∗∗^	62.72 ± 1.67^∗∗^	33.85 ± 1.96^∗∗^	14.01 ± 1.46^∗∗^
	25 *μ*mol·L^−1^	65.47 ± 3.49^∗∗^	79.67 ± 2.20^∗∗^	48.34 ± 3.52^∗∗^	34.07 ± 3.34^∗∗^
	12.5 *μ*mol·L^−1^	85.95 ± 4.63^∗∗^	88.75 ± 1.46^∗∗^	84.71 ± 1.94^∗∗^	61.30 ± 3.58^∗∗^

**Table 4:** Potential target proteins related to the signaling pathways involved in GBN.

Gene symbol	Uniprot_AC	Gene_ID	Target name	Score	PDB_pocket	Pathway
CDK2	P24941	1017	Cyclin-dependent kinase 2	-12.9	1jvp_P_LIG_301	PI3K/AKT, p53
HSP90AA1	P07900	3320	Heat shock protein HSP 90-alpha	-12	3d0b_A_SNX_233	PI3K/AKT
ABL1	P00519	25	Tyrosine-protein kinase ABL1	-11.5	2hyy_C_STI_600	ErbB
INS	P01308	3630	Insulin preproprotein	-11	2om1_e_RCO_1014	PI3K/AKT, mTOR, MAPK
MAPK14	Q16539	1432	Mitogen-activated protein kinase 14	-11	2zb1_A_GK4_361	MAPK
CSNK2A1	P68400	1457	Casein kinase II subunit alpha	-10.8	3r0t_A_FU9_338	Wnt
PPARD	Q03181	5467	Peroxisome proliferator-activated receptor delta	-10.6	1gwx_A_433_1	Wnt
PRKACA	P17612	5566	cAMP-dependent protein kinase catalytic subunit alpha isoform 1	-10.5	3l9l_B_L9L_351	Wnt, MAPK, hedgehog
EIF4E	P06730	1977	Eukaryotic translation initiation factor 4E	-10.5	4aza_A_MGO_1218	PI3K/AKT, mTOR
HSP90AB1	P08238	3326	Heat shock protein HSP 90-beta	-10.5	1uym_A_PU3_1224	PI3K/AKT
SRC	P12931	6714	Proto-oncogene tyrosine-proteinkinase Src	-10.4	2h8h_A_H8H_534	ErbB
IRAK4	Q9NWZ3	51135	Interleukin-1 receptor-associated kinase 4	-10.4	2nry_D_STU_501	MAPK
RXRA	P19793	6256	Retinoic acid receptor RXR-alpha	-10.3	1g5y_B_REA_501	PI3K/AKT
CHEK1	O14757	1111	Serine/threonine-protein kinase Chk1	-10.3	2e9n_A_76A_1001	p53
KDR	P35968	3791	Vascular endothelial growth factor receptor 2	-10.2	3vhe_A_42Q_1170	PI3K/AKT, MAPK
TGFBR1	P36897	7046	TGF-beta receptor type-1	-10.2	1py5_A_PY1_700	TGF-*β*, MAPK
MAPK10	P53779	5602	Mitogen-activated protein kinase 10	-10.1	2waj_A_SNB_1401	Wnt, MAPK, ErbB
BRAF	P15056	673	Serine/threonine-protein kinase B-raf	-10.1	4h58_A_10Z_801	ErbB, mTOR, MAPK

**Table 5:** The targets screened by GBN were consistent with the upregulated protein in colorectal cancer cells.

Item	Genes name
Upregulated	CDK2, THRB, RARA, MAOB, RARG, CTSS, AKR1D1, MMP3, NOS2, ACHE, ABL1, MAOA, AKR1C2, PTPN1, MMP12, PPARG, MMP13, CA2, MAPK14, INS, SEC14L2, PARP1, CSNK2A1, MMP8, PNLIP, PDE3B, RORC, Epn1, PPARD, AKR1C1, PDE4D, AKR1B1, HSP90AB1, CES1, BACE1, LCK, SRC, PPIA, TNK2, PKIA, CD1D, TTPA, ESR1, CHEK1, RXRA, NMRK1, OAT, PCTP, DCK, HMOX1, PIM1, KDR, C3, TGFBR1, GSR, RBP4, F10, REN, BRAF, MPO, DCXR, CD40LG, CASP7, FKBP1A

**Table 6:** Candidate targets screened by GBN.

Item	Genes' name
Candidate target	BRAF, TGFBR1, CSNK2A1, PPARD, CDK2, HSP90AB1, INS, KDR, RXRA, MAPK14, ABL1, SRC, CHEK1

**Table 7:** GBN_620 vs. c_SW620 differential protein GO classification statistics' table.

Cell line	Term type	GO term (GBN_620 vs. control differential protein number)
SW620	Biological process	Immune system process (22); biomodulation (97); metabolic process (76); intraspecific interactions between organisms(1);multiorganism process (1); transportation(4); reproductive process(14); cell process (112); development process (33); Multicellular organisms (12); interspecies interaction (9); grow (1); rhythmic process (1); localization (44); bioadhesion (6); behavior (2); signaling (2); biomineralization (1); stimulus response (51)
	Cell composition	Protein-containing complex (56); cellular anatomical entity (134)
	Molecular function	Transcription regulation activity (8); structural molecule activity (3); transport activity (9); molecular function regulation (15); binding (101); protein marker (1); molecular sensor (1); catalytic activity (54)

SW480	Biological process	Immune system process (7); biological regulation (26); metabolic process (33); transportation (2); reproductive process (4); cellular process (41); development process (10); multicellular biological process (7); interspecies interaction (6); growth (3); rhythmic process (1); localization (13); bioadhesion (1); behavior (1); signaling (1); stimulus response (16)
	Cell composition	Protein-containing complex (17); cellular anatomical entity (51)
	Molecular function	Structural molecular activity (6); transport activity (4); molecular function regulation (2); binding (36); catalytic activity (29)

**Table 8:** GBN vs. c_SW620 differential protein KEGG classification table (partial list).

No.	Pathway ID	Description	Number
1	hsa01100	Metabolic pathways	16
2	hsa00190	Oxidative phosphorylation	6
3	hsa05010	Alzheimer disease	6
4	hsa04714	Thermogenesis	5
5	hsa05016	Huntington disease	5
6	hsa05012	Parkinson disease	5
7	hsa04150	mTOR signaling pathway	4
8	hsa05200	Pathways in cancer	4
9	hsa05165	Human papillomavirus infection	4
10	hsa05163	Human cytomegalovirus infection	4
11	hsa01200	Carbon metabolism	3
12	hsa04141	Protein processing in endoplasmic reticulum	3
13	hsa04120	Ubiquitin-mediated proteolysis	3
14	hsa04115	p53 signaling pathway	3
15	hsa05131	Shigellosis	3
16	hsa05168	Herpes simplex virus 1 infection	3
17	hsa05166	Human T-cell leukemia virus 1 infection	3
18	hsa00051	Fructose and mannose metabolism	2
19	hsa00520	Amino sugar and nucleotide sugar metabolism	2
20	hsa01212	Fatty acid metabolism	2

**Table 9:** GBN vs. c_SW620 differential protein pathway classification statistics' table.

No.	First category	Second category	Number
1	Metabolism	Biosynthesis of other secondary metabolites	1
		Carbohydrate metabolism	5
		Energy metabolism	6
		Global and overview maps	16
		Glycan biosynthesis and metabolism	1
		Lipid metabolism	3
		Metabolism of cofactors and vitamins	1
		Metabolism of other amino acids	1
		Nucleotide metabolism	1
		Xenobiotics biodegradation and metabolism	1

2	Genetic information processing	Folding, sorting, and degradation	9
		Replication and repair	3
		Transcription	3
		Translation	5

3	Environmental information processing	Membrane transport	1
		Signal transduction	11

4	Cellular processes	Cell growth and death	6
		Cell motility	1
		Community-eukaryotes	3
		Transport and catabolism	3

5	Organismal systems	Circulatory system	1
		Digestive system	1
		Endocrine system	1
		Environmental adaptation	5
		Excretory system	1
		Immune system	6
		Nervous system	3

6	Human diseases	Cancer: overview	9
		Cancer: specific types	4
		Cardiovascular disease	1
		Drug resistance: antineoplastic	3
		Endocrine and metabolic disease	5
		Immune disease	3
		Infectious disease: cacterial	9
		Infectious disease: parasitic	2
		Infectious disease: viral	10
		Neurodegenerative disease	6
		Substance dependence	2

**Table 10:** GBN vs. c_SW480 differential protein KEGG classification table (partial list).

No.	Pathway ID	Description	Number
1	hsa01100	Metabolic pathways	5
2	hsa04210	Apoptosis	3
3	hsa01524	Platinum drug resistance	3
4	hsa05168	Herpes simplex virus 1 infection	3
5	hsa05163	Human cytomegalovirus infection	3
6	hsa05170	Human immunodeficiency virus 1 infection	3
7	hsa03013	RNA transport	2
8	hsa03010	Ribosome	2
9	hsa04150	mTOR signaling pathway	2
10	hsa04140	Autophagy-animal	2
11	hsa04145	Phagosome	2
12	hsa04714	Thermogenesis	2
13	hsa05132	Salmonella infection	2
14	hsa05131	Shigellosis	2
15	hsa05130	Pathogenic Escherichia coli infection	2
16	hsa05110	Vibrio cholerae infection	2
17	hsa00190	Oxidative phosphorylation	1
18	hsa00510	N-glycan biosynthesis	1
19	hsa00100	Steroid biosynthesis	1
20	hsa00564	Glycerophospholipid metabolism	1

**Table 11:** GBN vs. c_SW480 differential protein pathway classification statistics.

No.	First category	Second category	Number
1	Metabolism	Energy metabolism	1
		Global and overview maps	5
		Glycan biosynthesis and metabolism	1
		Lipid metabolism	2
		Metabolism of cofactors and vitamins	1
		Metabolism of terpenoids and polyketides	1

2	Genetic information processing	Folding, sorting, and degradation	3
		Replication and repair	1
		Transcription	1
		Translation	4

3	Environmental information processing	Signal transduction	2
		Signaling molecules and interaction	1

4	Cellular processes	Cell growth and death	4
		Cellular community-eukaryotes	2
		Transport and catabolism	5

5	Organismal systems	Endocrine system	2
		Environmental adaptation	2
		Excretory system	2
		Immune system	3
		Nervous system	2

6	Human diseases	Cancer: overview	2
		Cancer: specific types	2
		Cardiovascular disease	1
		Drug resistance: antineoplastic	3
		Endocrine and metabolic disease	2
		Immune disease	1
		Infectious disease: bacterial	6
		Infectious disease: parasitic	2
		Infectious disease: viral	5
		Neurodegenerative disease	1

## Data Availability

The data that support the findings of this study are available from the corresponding authors upon reasonable request. Some data may not be made available because of privacy or ethical restrictions.
